# The cardiomyopathy of cystic fibrosis: a modern form of Keshan disease

**DOI:** 10.3389/fcvm.2024.1285223

**Published:** 2024-02-01

**Authors:** Javier Segovia-Cubero, Lorena Ruiz-Bautista, Luis Maiz-Carro, Rosa M. Girón-Moreno, M. Concepción Prados-Sánchez, M. Teresa Martínez-Martínez, Montserrat González-Estecha, Susana Mingo-Santos, Manuel Gómez-Bueno, Clara Salas-Antón, Miguel A. Cavero-Gibanel, Miguel Pastrana-Ledesma, Pablo García-Pavía, Rosalía Laporta-Hernández, David Sánchez-Ortiz, Luis Alonso-Pulpón

**Affiliations:** ^1^ Cardiolology Dept., Hospital Universitario Puerta de Hierro, Madrid, Spain; ^2^ Centro de Investigación Biomédica en Red en Enfermedades Cardiovasculares (CIBERCV), Madrid, Spain; ^3^ Cystic Fibrosis Unit, Pneumology Dept., Hospital Universitario Ramon y Cajal, Madrid, Spain; ^4^ Cystic Fibrosis Unit, Pneumology Dept., Hospital Universitario La Princesa, Madrid, Spain; ^5^ Cystic Fibrosis Unit, Pneumology Dept., Hospital Universitario La Paz, Madrid, Spain; ^6^ Cystic Fibrosis Unit, Pneumology Dept., Hospital Universitario Doce de Octubre, Madrid, Spain; ^7^ Trace Element Laboratory, Biochemistry Dept., Hosp. Universitario San Carlos, Madrid, Spain; ^8^ Pathology Dept., Hospital Universitario Puerta de Hierro, Madrid, Spain; ^9^ Radiology Dept., Hospital Universitario Puerta de Hierro, Madrid, Spain; ^10^ Pneumology Dept., Hospital Universitario Puerta de Hierro, Madrid, Spain

**Keywords:** cystic fibrosis, cardiomyopathy, selenium deficiency, Keshan disease, heart failure

## Abstract

**Introduction:**

We conducted a study to determine the prevalence of structural heart disease in patients with CF, the characteristics of a cardiomyopathy not previously described in this population, and its possible relationship with nutritional deficiencies in CF.

**Methods:**

We studied 3 CMP CF patients referred for heart-lung transplantation and a prospective series of 120 adult CF patients. All patients underwent a clinical examination, blood tests including levels of vitamins and trace elements, and echocardiography with evaluation of myocardial strain. Cardiac magnetic resonance imaging (CMR) was performed in patients with CMP and in a control group. Histopathological study was performed on hearts obtained in transplant or necropsy.

**Results:**

We found a prevalence of 10% (CI 4.6%–15.4%) of left ventricular (LV) dysfunction in the prospective cohort. Myocardial strain parameters were already altered in CF patients with otherwise normal hearts. Histopathological examination of 4 hearts from CF CMP patients showed a unique histological pattern of multifocal myocardial fibrosis similar to Keshan disease. Four of the five CF CMP patients undergoing CMR showed late gadolinium uptake, with a characteristic patchy pattern in 3 cases (*p* < 0.001 vs. CF controls). Selenium deficiency (Se < 60 µg/L) was associated with more severe LV dysfunction, higher prevalence of CF CMP, higher NTproBNP levels, and more severe pulmonary and digestive involvement.

**Conclusion:**

10% of adults with CF showed significant cardiac involvement, with histological and imaging features resembling Keshan disease. Selenium deficiency was associated with the presence and severity of LV dysfunction in these patients.

## 1 Introduction

Cystic fibrosis (CF) represents the most common lethal hereditary disorder in individuals of Caucasian descent, with an incidence ranging from 1 in 2,000 to 1 in 5,000 live births. The condition arises from mutations in the CFTR gene, which codes for the Cystic Fibrosis Transmembrane Conductance Regulator—a key protein facilitating the movement of chloride ions and other electrolytes across cellular membranes (1). Prior to the substantial progress in treatment over the past two decades, most individuals with CF succumbed to the disease in their youth. However, recent advancements have markedly prolonged the life span of those affected, now potentially surpassing 40 years (2, 3). Continuous development in targeted therapies is expected to yield further enhancements in patient outcomes shortly (1, 3).

Historically, cardiac involvement has not been predominantly recognized within the spectrum of CF pathology. Although cor pulmonale due to chronic hypoxemia was once suggested to be present in advanced stages of CF lung disease, this has not been substantiated by rigorous research (3–5). Nonetheless, comprehensive autopsies of young CF patients have documented pronounced cardiac abnormalities, including left ventricular enlargement and a distinctive histological presentation termed multifocal myocardial fibrosis (MMF) (6, 7). Similar cardiac histological patterns have been observed in individuals with severe malabsorption disorders (8), and intriguingly, in cases of Keshan disease (9) —a region-specific cardiomyopathy in parts of China predominantly affecting children and pregnant females, linked to selenium deficiency, and largely eradicated following Se supplementation in at-risk groups (10–12).

Prompted by the identification of Se deficiency in a CF patient evaluated for heart-lung transplantation, we structured a prospective study aimed at assessing the incidence of structural heart disease within the CF demographic. This research also uncovers the features of a previously unidentified cardiac condition and explores its potential association with the nutritional complications inherent to CF. The importance of this work lies in the need to clarify the existence of this entity and its manifestations, establish the appropriate diagnostic tools and suggest pathophysiological hypotheses that could help improve the prevention of its appearance and treatment in the future.

## 2 Materials and methods

We recruited a series of adult CF patients without known cardiac disease from all of the four specific CF outpatient clinics in our health area. We also analyzed the characteristics of the patients referred for HLTx who motivated the project. We obtained the approval of the Research Ethics Committees of the five participating institutions. Informed consent was obtained from patients and controls.

Clinical data were obtained by structured interview. Patients were grouped according to the presence of homozygous or heterozygous F508del mutation of CFTR gene or other mutations. Pancreatic insufficiency was stratified by dose of pancreatic enzyme supplements (thresholds 1,000 and 2,000 UI). For pulmonary involvement, the presence and type of pulmonary colonization were recorded, as well as the severity of pulmonary disease expressed as % of FEV1 and other spirometry parameters.

Blood tests: Blood samples were collected after an 8 h overnight fasting period and were processed at the same laboratory. Trace element samples were processed in accordance with the Guideline of the Clinical and Laboratory Standards Institute, as shown in Supplementary Appendix I of Supplementary Material.

Cardiac evaluation: Twelve-lead surface EKG and an echocardiographic study (IE33 digital ultrasound system®, Philips Medical Systems, Amsterdam, Holland) were performed. Echocardiographic evaluation was performed according to the American Society of Echocardiography (ASE) recommendations as shown in Supplementary Appendix II (13).

Established CF cardiomyopathy was defined by the presence of a left ventricular ejection fraction (LVEF) ≤ 50% (systolic dysfunction) without other obvious cause in cardiac study. LV dysfunction was diagnosed in the presence of either systolic or diastolic dysfunction. LV longitudinal and circumferential strain measurements were performed by speckle tracking technique according to the EACVI/ASE recommendations (14). A series of 21 healthy adults matched for age and gender served as a control group for strain and strain-rate data comparisons. A reproducibility analysis of myocardial deformation measures was performed and is available in Supplementary Appendix II.

In order to define the characteristics of CF cardiomyopathy we studied a group of 8 patients including 3 patients referred to our center for HLTx and 5 patients with cardiomyopathy (systolic dysfunction) detected in the prospective cohort. Cardiac magnetic resonance (CMR) and histology when possible (organs explanted at HLTx) were carried out in this group. CMR was performed with a 1.5 T equipment, (Philips Intera CV, Best, The Netherlands®). A subgroup of 20 volunteer CF individuals without cardiac dysfunction served as control. CMR protocol is described in Supplementary Appendix III of Supplemental Material.

Statistical analysis: Shapiro-Wilk test was used to confirm the normal distribution hypothesis. Comparison between groups was performed with the Student's t test for independent samples with a normal distribution and the Mann-Whitney test for non-normally distributed variables. The strain values were contrasted by the one-way ANOVA test and the post-hoc multiple comparison Tukey test. The comparison of two proportions in independent samples was performed using the *χ*^2^ test and Fisher's exact test as appropriate. Risk was estimated with the OR and 95% CI. A two-sided *p*-value less than 0.05 was considered statistically significant. All analyses were performed with SPSS® v21 (SPSS Inc., Chicago, IL, USA).

## 3 Results

### 3.1 Cardiomyopathy associated with CF

In this study, we delineate a cohort of 8 patients presenting with a distinct form of cardiomyopathy, which we propose to term “Cardiomyopathy of Cystic Fibrosis” (CF CMP) henceforth. Among these individuals, 3 were referred to our institution for heart-lung transplantation (HLTx), while the remaining 5 were identified through our prospective study, exhibiting a left ventricular ejection fraction (LVEF) below 50%. Table 1A shows the clinical attributes of CF in these subjects, comparing them with CF patients who do not present with CMP. Overall, those with CMP demonstrated exacerbated respiratory and gastrointestinal manifestations of CF, in addition to lower body mass indices, frequently registering as underweight. Genetic analysis of patients disclosed the following mutations in the CFTR gene: three subjects possessed a homozygous F508del mutation, one a heterozygous F508del mutation, one a homozygous G542X mutation, one a homozygous S549R mutation, and two exhibited no identifiable mutations.

**Table 1A:** Comparison of the characteristics of CF patients with cardiomyopathy with those of CF patients with normal hearts.

	CF with CMP (*n* = 8)	CF without CMP (*n* = 115)	*p* value
Male gender (%)	63%	51%	0.72
Age (years, mean ± SD)	29.2 ± 6.4	31.1 ± 8.9	0.5
Late diagnosis (%)	14%	21%	0.5
CFTR gene mutation			0.2
508del Homozygous (%)	38%	29%	
508del Heterozygous (%)	13%	35%	
Other mutations	25%^a^	17%	
Unknown	25%	19%	
Pancreatic insufficiency (%)	100%	79%	0.1
High dose of PES (%)	63%	33%	0.01
BMI(kg/m^2^) (mean ± SD)	19.3 ± 3.2	22.0 ± 3.1	0.01
BMI < 20 (kg/m^2^) (%)	75%	26%	0.006
FEV_1_% (mean ± SD)	36.5 ± 20	61 ± 22	0.02
FEV_1 _< 40% (%)	63%	22%	0.04
Airway colonization (%)	100%	93%	0.9
Pseudomonas colonization (%)	100%	56%	<0.001
Echo LVEDD (mm, mean ± SD)	61 ± 6	44 ± 5	<0.001
Echo LVEF (%, mean ± SD)	37 ± 10	65 ± 6	<0.001
Echo LV Tei index (mean ± SD)	0.6 ± 0.2	0.4 ± 0.1	0.003
Echo TAPSE (mm, mean ± SD)	19.5 ± 3	22 ± 4	0.2
Echo diastolic dysfunction (%)	38%	6%	0.02
MMF in cardiac MR (%)^b^	60% (3/5)	0 (0/20)	<0.001

^a^ 2 patients with G542X homozygous and S549R homozygous mutations of CFTR gene.

^b^ 5 patients in the CMP group and 20 patients in the non-CMP group underwent cardiac MR.

CF, cystic fibrosis; CMP, cardiomyopathy; CFTR, cystic fibrosis transmembrane conductance regulator FEV1: forced espiratory volumen in first second; PES, pancreatic enzymes supplements; BMI, body mass index; Echo, echocardiography; LVEDD, left ventricular end diastolic dimension; LVEF, left ventricular ejection fraction; LV, left ventricle; TAPSE, tricuspid annular plane systolic excursion; MFF, multifocal fibrosis; MR, magnetic resonance.

Laboratory findings, as delineated in Table 1B, revealed salient disparities such as elevated NT-proBNP levels and a pronounced occurrence of selenium (Se) deficiency within the CF CMP cohort, though aggregate Se concentrations remained analogous across both patient groups.

**Table 1B:** Selected laboratory values of CF patients with cardiomyopathy as compared with those without it.

	CF with CMP (*n* = 8) (mean ± SD)	CF without CMP (*n* = 115) (mean ± SD)	*p* value
Leukocytes (cels × 10^3^/ml)	8.2 ± 1.5	8.5 ± 3.1	0.7
Neutrophils (%)	72 ± 6	63 ± 9	0.4
Lymphocytes (%)	18 ± 2	26 ± 8	0.3
Hemoglobin (g/dl)	14.3 ± 1.2	14.2 ± 1.5	0.2
Platelets (cels × 10^3^/ml)	311 ± 62	309 ± 103	0.7
Prothrombin time (sec)	11.6 ± 1.7	11.4 ± 1,0	0.6
APTT (sec)	3.7 ± 3.0	30.2 ± 3.3	0.6
Glucose (g/dl)	87 ± 10	97 ± 22	0.11
Creatinine (mg/dl)	1.07 ± 0.3	0.95 ± 0.13	0.04
Urea (mg/dl)	35 ± 30	31 ± 8	0.3
Total Cholesterol (mg/dl)	131 ± 44	162 ± 46	0.4
LDL-cholesterol (mg/dl)	72 ± 35	92 ± 36	0.7
HDL-cholesterol (mg/dl)	43 ± 9	51 ± 14	0.2
Triglycerides (mg/dl)	81 ± 41	100 ± 44	0.8
AST (U/L)	25 ± 8	25 ± 9	1
ALT (U/L)	34 ± 22	25 ± 13	0.5
GGT (U/L)	58 ± 38	27 ± 30	0.6
NTproBNP (pg/ml)^a^	75 (33–429)	52 (25–79)	<0.01
Iron (µg/dl)	60 ± 53	71 ± 35	0.8
Ferritin (ng/ml)	125 ± 111	82 ± 75	0.14
Transferrin (mg/dl)	283 ± 73	279 ± 51	1
Transferrin saturation index (%)	23 ± 16	21 ± 10	0.5
Vitamin A (µg/ml, normal 0.4–0.8)	0.43 ± 0.18	0.40 ± 0.17	0.6
Vit. A/Retinol Binding Prot. (nl3.5–7.5)	0.78 ± 0.13	0.60 ± 0.34	0.2
Vitamin E (µg/ml, normal 8–21)	9.6 ± 2.6	10.5 ± 3.7	0.3
Vitamin E/Total cholesterol (nl 6–12)	5.6 ± 1.4	6.5 ± 2.1	0.2
Vitamin D (ng/ml, normal 20–50)	18.0 ± 4.7	26.2 ± 13.5	0.05
Vitamin B12 (pg/ml, normal 197–866)	938 ± 560	704 ± 293	0.7
Vitamin B9 (ng/dl, normal 3.1–17.5)	12 ± 8	14 ± 7	0.4
Selenium (µg/L, normal range 60–120)	71 ± 18	72 ± 15	0.9
Selenium <60 µg/dl (%)	63%	16%	0.006
Copper (µg/L, normal range 75–150)	117 ± 21	133 ± 39	0.1
Zinc (µg/L, normal range 60–150)	90 ± 9	86 ± 14	0.3
Mercury^a^ (µg/L, normal range <10)	5.6 (0.5–10)	5.7 (3–9)	1
Lead^a^ (µg/L, normal range < 1)	0.6 (0.5–1)	0.8 (0.4–1)	0.7

^a^ Non-normally distributed variables are expressed as Median (interquartile range).

CF, cystic fibrosis; CMP, cardiomyopathy; APTT, activated plasma thromboplastin time; AST,  aspartate aminotransferase; ALT, alanin aminotransferase; GGT, gamma-glutamyl transpeptidase; Nt-proBNP, N-terminal pro B-type natriuretic peptide.

Electrocardiography in patients with CMP uniformly showed normal sinus rhythm with standard PR and QTc intervals. Two EKG showed signs of left atrial enlargement, two displayed intraventricular conduction delays, and five presented repolarization anomalies suggestive of LV overload. Chest radiography consistently confirmed signs of CF-related pulmonary involvement in all cases, yet an augmented cardiothoracic index, devoid of associated pulmonary congestion, was visible in only two of the eight patients.

Echocardiographic measures for the CF CMP subjects (Table 1A), principally highlighted notable discrepancies in LV function relative to CF patients without CMP, while parameters of right ventricular function, such as Tricuspid Annular Plane Systolic Excursion (TAPSE), were comparable between both groups. Moreover, a substantial decrease in longitudinal strain values was observed (−13 ± 6 for CF CMP vs. −20 ± 5 for CF patients without CMP, *p* = 0.04). Within the CMP subset, only a singular patient exhibited mitral regurgitation of a moderate degree. Fundamentally, echocardiographic findings from the CF CMP population resembled those associated with other dilated cardiomyopathy variants.

**Figure 1:**
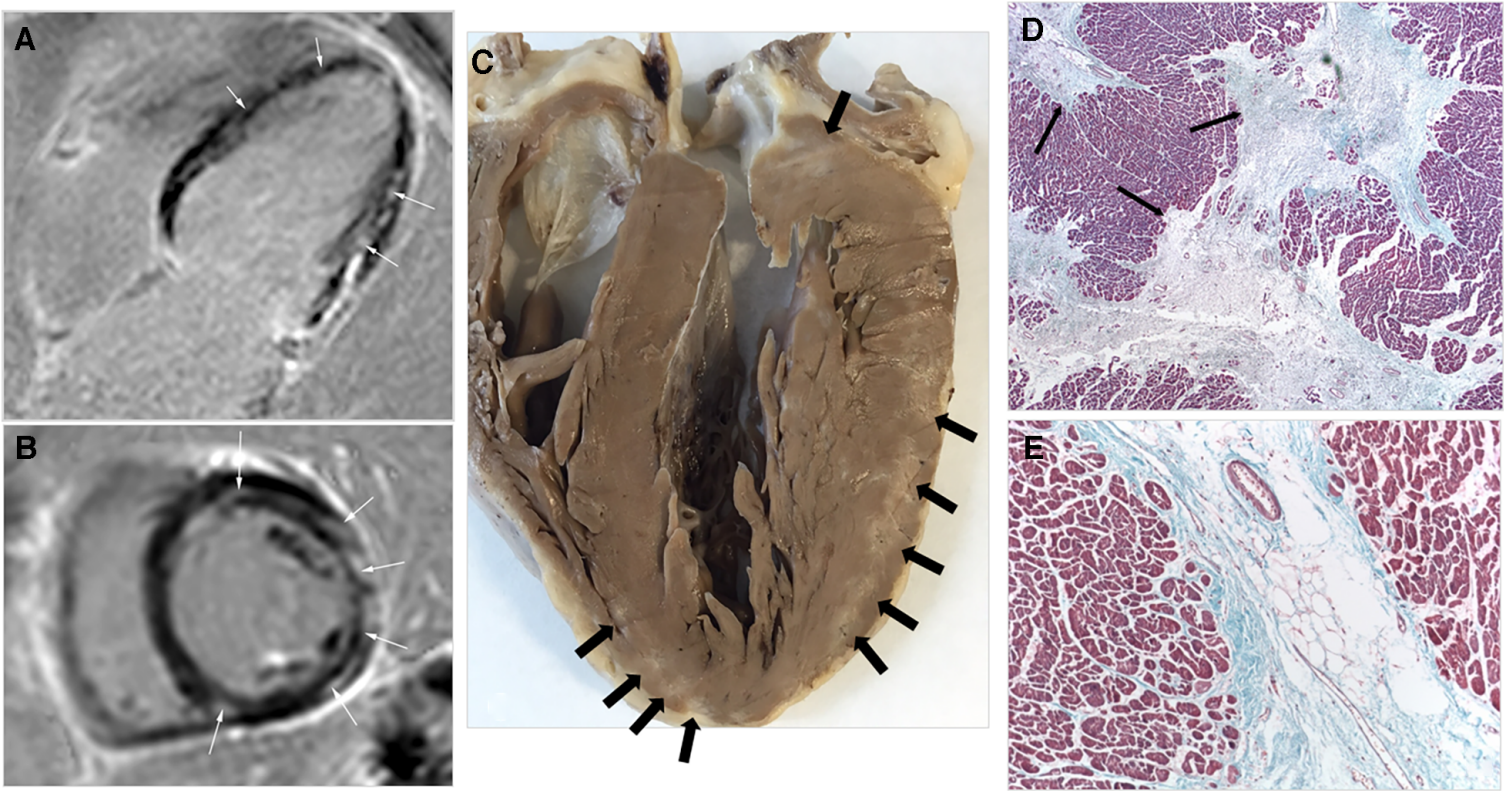
(**A,B**) CMR imaging in a patient with CF cardiomyopathy. Late gadolinium enhancement PSIR (phase sensitive inversion recovery) sequences seen on (**A**) long axis four-chamber view and (**B**) short axis view. Multiple patchy fibrotic areas of late gadolinium enhancement are evident, with a fibrosis pattern that is neither transmural nor subendocardial (small arrows). (**C–E**) Pathology of CF CMP. (**C**) Macroscopic view of a four-chamber section of the entire heart explanted during cardiopulmonary transplantation. Whitish areas of patchy fibrosis are evident within the myocardium of the free wall of the LV and the apical region of the interventricular septum (arrows). (**D**) Myocardium from LV wall showing multifocal myocardial fibrosis (MMF): large bands of lax fibrotic tissue are interspersed with areas of myocardium. (Masson trichrome staining, original magnification 40×). (**E**) Increased magnification (original 200×), showing lack of inflammatory cells at the grossly preserved myocardial areas, with atrophic myocardial fibers encroached by surrounding fibrotic tissue at the edges.

Cardiac MRI, conducted on five CMP subjects (all CMP patients excluding the 3 who received HLTx), revealed late gadolinium enhancement in four patients, with three demonstrating a patchy myocardial uptake dispersed arbitrarily across the LV walls (Figure 1). Another patient displayed marginal linear subepicardial gadolinium uptake, while the one with a higher LVEF (48%) showed no detectable enhancement. In contrast, gadolinium uptake was absent in CMR studies of 20 CF patients with an unremarkable echocardiogram (*p* < 0.001 by comparison).

**Figure 2:**
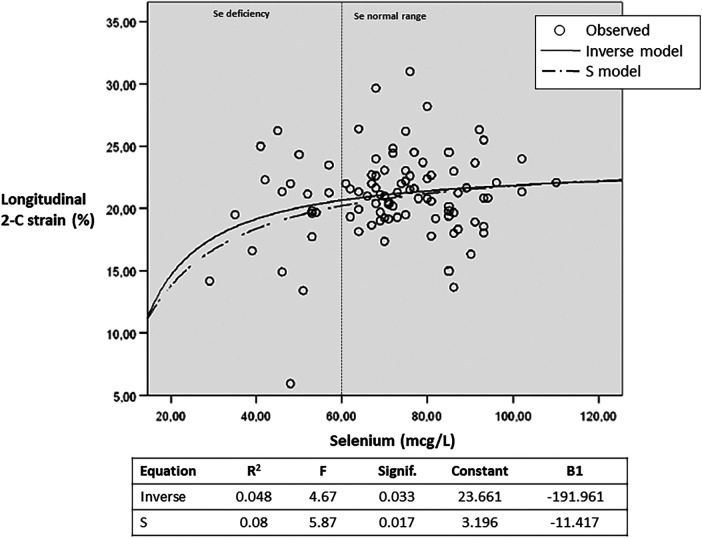
Non-linear models (inverse and S) of correlation between Se levels and longitudinal 2-chamber strain values. The models reflect stability of LV contractility values when Se levels are >60 mcg/L, and a tendency to progressively lower values below this cut-off Se level. Equations: Inverse: y = b0 + (b1/t) and S: y = eE(b0 + b1/t), where y = strain 2C; b0 = constant; b1 = B1; t = Se level.

Pathologically, the four hearts available for examination (three explanted during HLTx and one necropsy from a CMP patient who succumbed to pulmonary infection) showcased congruent findings: Macroscopic inspection of sections of LV walls revealed patchy, whitish scars interspersed within otherwise normal myocardium (Figure 2A). Histological scrutiny identified the distinctive patterns of multifocal myocardial fibrosis (MMF), characterized by bands of fibrosis weaving through largely intact myocardial territories (Figures 2B,C). It is imperative to note the absence of inflammatory cellular infiltration or disruption of the myocardial architecture. The collagen within the fibrotic strands presented a characteristic lax appearance, distinctly differing from scarring consequent to ischemic necrosis. Coronary arteries were normal in all inspected cases.

### 3.2 Prevalence of left ventricular dysfunction in adults with cystic fibrosis

Out of 320 adults attending four specialized cystic fibrosis outpatient clinics, 135 agreed to participate in our prospective study and provided informed consent. Fifteen of these participants were unable to complete the necessary evaluations and were subsequently excluded from the analysis, resulting in a cohort of 120 consecutively enrolled, unselected adult CF patients devoid of any known cardiac pathologies. It is noteworthy to mention that none of the patients were receiving CF transmembrane conductance regulator modulator therapies, including lumacaftor, ivacaftor, tezacaftor, or elexacaftor, as the data collection predated the introduction of these treatments in our country.

Echocardiographic assessments identified left ventricular (LV) dysfunction in 12 individuals [10%; 95% Confidence Interval (CI): 4.6%–15.4%]. Among these, five (4.2%, CI: 0.6%–7.8%) were found to have significant systolic LV dysfunction: two (1.7%) exhibited isolated systolic impairment, while three (2.5%) presented with concurrent systolic and diastolic dysfunction. An additional seven participants (5.8%, CI: 1.6%–10%) were diagnosed with isolated diastolic LV dysfunction. Comprehensive clinical, laboratory, and echocardiographic parameters for the total study population and its respective subgroups are organized in Tables 2A,B.

**Table 2A:** Clinical and cardiac and echocardiographic data of patients in the prospective cohort and its subgroups.

	Prospective cohort (*n* = 120)	CF without LV dysfunction (group A, *n* = 108)	CF with LV dysfunction (group B, *n* = 12)	*p* value (A vs. B)
Male gender (%)	53%	50%	83%	0.06
Age (years, mean ± SD)	31 ± 9	31 ± 9	32 ± 8	0.6
Age at CF diagnosis (years, mean ± SD)	11 ± 14	11 ± 14	8 ± 10	0.9
Genetics (CFTR mutations)				0.3
508del Homozygous	29%	30%	17%	
508del Heterozygous	34%	36%	17%	
Other mutations	18%	15%	41%	
Unknown	19%	18%	25%	
Pancreatic insufficiency (%)	80%	79%	83%	0.5
Diabetes (%)	21%	20%	25%	0.7
Hepatic disease (%)	40%	36%	75%	0.02
Pancreatic enzymes supplements				0.06
>200,000 units/day (lipase)	35%	31%	67%	
100,000–200,000 units/day	45%	48%	17%	
<100,000 units/day	20%	20%	17%	
Need for nutritional supplements				
Vitamin A	46%	44%	58%	0.4
Vitamin E	80%	78%	100%	0.12
Polivitamins	67%	65%	83%	0.3
BMI (kg/m^2^) (mean ± SD)	22.0 ± 3.3	22.6 ± 3.2	19.1 ± 3.8	0.03
BMI < 20 (kg/m^2^) (%)	29%	24%	75%	<0.01
%FEV_1_ (mean ± SD)	60 ± 22	61 ± 23	51 ± 18	0.09
FEV_1 _< 40% (%)	23	21	58	0.02
Chronic airway colonization (%)	92%	93%	83%	0.2
*Pseudomonas aeruginosa. (%)*	58%	54%	100%	<0.01
*Staph. aureus* (%)	50%	52%	33%	0.4
Echocardiographic data				
Aortic root (mm)	26.8 ± 3.6	26.3 ± 3.2	29.6 ± 4.8	0.001
Left atrium (mm)	29.8 ± 4.3	29.5 ± 3.8	32.1 ± 6.2	0.02
Interventricular septum (mm)	8.6 ± 1.8	8.6 ± 1.8	8.5 ± 2.2	0.9
LV mass (g)	133 ± 51	127.6 ± 44.5	163.4 ± 81	0.01
LVEDD (mm)	44.3 ± 6.0	44.0 ± 5.0	48.7 ± 10.3	0.002
LVEF (%)	64.0 ± 7.9	65.0 ± 6.0	51.3 ± 11	0.4
Mitral E wave vel. (cm/s)	82 ± 13	82 ± 12	82 ± 21	0.9
E/A wave ratio	1.3 ± 0.3	1.3 ± 0.3	1.3 ± 0.4	0.8
Deceleration Time E wave (ms)	194 ± 35	192 ± 32	212 ± 52	0.4
E/e’ ratio	6.1 ± 1.8	6.0 ± 1.0	6.9 ± 3.5	0.06
Isovolumic relaxation time (ms)	63 ± 12	61 ± 11	64.3 ± 12	0.5
LV Tei index	0.5 ± 0.1	0.5 ± 0.1	0.6 ± 0.1	0.002
TAPSE (mm)	21.7 ± 4.2	21.0 ± 3.0	20.3 ± 3.0	0.2
eSPAP (mm Hg)	29.2 ± 10.7	28.8 ± 9.6	31.5 ± 17	0.5
Inferior vena cava (mm)	11.5 ± 3.1	10.4 ± 2.9	11.4 ± 2.0	0.9

CF, cystic fibrosis; LV, left ventricular; BMI, body mass index; %FEV_1_, percent Forced expiratory volume in the first second; LVEDD, left ventricular end diastolic dimension; LVEF, left ventricular ejection fraction; TAPSE, tricuspid annular plane systolic excursion; eSPAP, estimated systolic pulmonary artery pressure.

**Table 2B:** General laboratory results, vitamin and trace elements of patients in the prospective cohort and its subgroups.

	Prospective cohort (*n* = 120)	CF without LV dysfunction (group A, *n* = 108)	CF with LV dysfunction (group B, *n* = 12)	*p* value (A vs. B)
Hemoglobin (g/dl)	14.2 ± 1.5	14.1 ± 1.4	15.0 ± 1.3	0.1
Leucocytes (10^3^/µl)	8.5 ± 3.0	8.2 ± 2.8	10.4 ± 3.1	0.008
Neutrophils (%)	63.3 ± 9.0	62.2 ± 8.6	71.4 ± 6.1	0.001
Lymphocytes (%)	25.9 ± 8.0	26.9 ± 7.8	18.5 ± 5.2	0.00
Platelets (10^3^/µl)	308.1 ± 99.4	306.9 ± 91.8	315.3 ± 144	0.8
Prothrombin activity (%)	102.4 ± 12.0	102.6 ± 11.3	99.4 ± 15.6	0.3
APTT (s)	30.3 ± 3.3	30.2 ± 3.4	30.4 ± 2.0	0.8
Glucose (g/dl)	95.8 ± 21.0	94.4 ± 15.5	104.7 ± 42	0.08
Creatinine (mg/dl)	0.89 ± 0.12	0.88 ± 0.16	1.1 ± 0.20	0.04
Urea (mg/dl)	32.1 ± 12.7	30.8 ± 7.7	40.8 ± 28.6	0.07
Total Cholesterol (mg/dl)	162.8 ± 45.7	165.0 ± 47.7	174.7 ± 42	0.2
LDL-cholesterol (mg/dl)	93.9 ± 35.7	95.2 ± 36.9	106.6 ± 22	0.3
HDL-cholesterol (mg/dl)	50.1 ± 14.0	51.2 ± 14.3	42.2 ± 8.2	0.03
Triglycerides (mg/dl)	101.7 ± 43.9	101.6 ± 45.6	110.6 ± 30.9	0.5
AST (U/L)	25.4 ± 9.0	24.8 ± 9	29.1 ± 8.6	0.1
ALT (U/L)	26.9 ± 23.5	26.4 ± 24.5	29.6 ± 14	0.6
GGT (U/L)	27.4 ± 29.8	24.2 ± 21.7	50.7 ± 60	0.003
Ceruloplasmin (mg/dl)	34 ± 9.1	34.3 ± 9	31.2 ± 9.5	0.3
Iron (µg/dl)	71.5 ± 35.1	70.6 ± 33.2	76.8 ± 46.7	0.5
Ferritin (ng/ml)	88.5 ± 45	86.2 ± 80	98.0 ± 71.7	0.9
Transferrin (mg/dl)	277 ± 51	276 ± 51	283 ± 49	0.6
Transf. saturation index (%)	20.8 ± 10.9	20.5 ± 9.7	22.9 ± 17.2	0.5
NTproBNP (pg/ml)^a^	144.5 ± 802.2	55 ± 35.7	694.5 ± 2124	0.01
Creatin kinase (IU/L)	104.2 ± 120.1	97.2 ± 91.1	153.7 ± 247.8	0.1
C reactive protein (mg/L)	9.0 ± 10.1	9.3 ± 10.3	6.3 ± 5.3	0.5
Vitamins				
Vitamin A (µg/ml, normal 0.4–0.8)	0.40 ± 0.20	0.40 ± 0.18	0.43 ± 0.26	0.9
Vit. A/Retinol Binding Prot. (3.5–7.5)	0.60 ± 0.32	0.59 ± 0.34	0.76 ± 0.18	0.1
Vitamin E (µg/ml, normal 8–21)	10.4 ± 3.6	10.5 ± 3.7	7.9 ± 2.1	0.01
Vitamin E/Total cholesterol (nl 6–12)	6.4 ± 2.0	6.5 ± 2.1	5.5 ± 1.9	0.1
Vitamin D (ng/ml, normal 20–50)	25.3 ± 13.5	26.2 ± 13.3	16.7 ± 7.1	0.01
Vitamin B12 (pg/ml, normal 197–866)	714.6 ± 304	702 ± 298	829 ± 406	0.1
Vitamin B9 (ng/dl, normal 3.1–17.5)	13.6 ± 6.6	14 ± 7.9	13.4 ± 7.2	0.9
Trace elements				
Selenium (µg/L, normal 60–120)	72 ± 15	72 ± 15	71 ± 18	0.8
Selenium <60 µg/dl (%)	17	16	60	0.03
Copper (µg/L, normal 75–150)	131 ± 37	133 ± 38	118 ± 18	0.06
Zinc (µg/L, normal 60–150)	87 ± 13.5	86 ± 14	91 ± 10	0.2
Mercury^a^ (µg/L, normal < 10)	5.7 (3–10)	5.8 (3–9)	5.6 (0.5–9.5)	0.8
Lead^a^ (µg/L, normal < 1)	0.8 (0.4–1)	0.8 (0.4–1)	1.0 (0.5–1)	0.5

^a^ Non-normally distributed variables are expressed as Median (interquartile range).

APTT, activated plasma thromboplastin time; AST, aspartate aminotransferase; ALT, alanin aminotransferase; GGT, gamma-glutamyl transpeptidase; NTproBNP, N-terminal pro b-type natriuretic peptide.

Table 3 details the assessment of myocardial deformation parameters. Imaging quality impeded the deformation analysis of six patients, all pertaining to the subgroup with no LV dysfunction, thus reducing our sample to 114. The collective myocardial deformation measures among the CF patient cohort generally resided within normal parameters. Nonetheless, these figures were significantly inferior to those of a control group consisting of 21 healthy volunteers, suggesting that even CF patients with a standard cardiac evaluation may present subtly compromised myocardial mechanics.

**Table 3:** Myocardial deformation analysis in the prospective series. Comparison of average strain and strain-rate values between normal controls, CF patients with normal LV function and CF patients with LV dysfunction of any type.

	Control group (*n* = 21)	CF without LV dysfunction (group A, *n* = 102)	CF with LV dysfunction (group B, *n* = 12)	*p* value Controls vs. A	*p* value Controls vs. B	*p* value A vs. B
C S (%)	−22.1 ± 3.8	−19.7 ± 3.8	−17.4 ± 5.5	0.04	0.003	0.03
C SR	−1.5 ± 0.4	−1.3 ± 3.8	−0.9 ± 0.3	0.04	0.003	0.004
4C S (%)	−22.2 ± 2.9	−20.7 ± 2.8	−17.4 ± 5.2	0.09	0.001	<0.005
4C SR	−1.4 ± 0.3	−1.2 ± 0.3	−0.9 ± 0.3	0.28	0.001	<0.005
2C S (%)	−22.9 ± 3.3	−21.4 ± 3.6	−15.8 ± 4.6	0.28	0.001	<0.005
2C SR	−1.3 ± 0.2	−1.3 ± 0.3	−0.9 ± 0.4	0.7	0.001	<0.005

S, strain; SR, strain-rate; C, circumferential strain; C, circumferential strain-rate; 4C, four-chamber longitudinal parameters; 2C, two-chamber longitudinal parameters.

When focusing on CF patients with LV dysfunction, the myocardial deformation indices were substantially below normative values, showing significant reductions compared to both healthy controls and CF patients without LV impairment. Further analysis within the LV dysfunction classification revealed the most pronounced anomalies in patients with systolic dysfunction; comparative examination against those with solely diastolic dysfunction approached statistical significance in the case of longitudinal strain (−14.7 ± 4.2% vs. −19.5 ± 5.1%, *p* = 0.05).

### 3.3 Nutritional factors associated with cystic fibrosis cardiomyopathy

Vitamin levels in the prospective cohort and in CMP patients are shown in Table 2B. As anticipated within a population receiving tailored oral supplementation, average vitamin levels were predominantly in the normal range. However, trends toward lower concentrations of vitamins E and D were observed in CMP patients (*p* = 0.09 and *p* = 0.05, respectively). Vitamin D deficiency was more prevalent in the CMP subset (38%) compared to the broader CF cohort (17%), though this did not reach statistical significance (*p* = 0.17). Notably, we found no substantial correlations between vitamin levels and indices of left ventricular function.

The trace element profiles are summarized in Table 2B, with Cadmium levels excluded due to most (>80%) patients having measurements below detectable thresholds. Overall, trace element values in CF patients fell within acceptable ranges. Although the mean selenium (Se) levels did not differ significantly between those with and without LV dysfunction, Se deficiency (Se < 60 µg/L, identified in 17.5% of the prospective series) was linked to lower LVEF, an increased prevalence of LV dysfunction and CMP (14% among Se-deficient patients vs. 2% in the remainder, *p* = 0.037), as well as heightened NT-proBNP concentrations (Table 4). Furthermore, Se deficiency was associated with deleterious myocardial deformation metrics, higher instances of undernourishment indicated by BMI < 20 kg/m^2^, and more severe pulmonary disease manifestations. No relationships were found between LVEF and other trace elements.

**Table 4:** Comparison of selected clinical, laboratory and echocardiographic values between Se-deficient CF patients and CF patients with normal Se blood levels.

	Normal Se (*n* = 99)	Se deficient (*n* = 21)	*p* value
Blood Selenium (mcg/L, mean ± SD)	77 ± 10	48 ± 7	
Male gender (%)	50	52	1
Age (years, mean ± SD)	32 ± 9	29 ± 11	0.2
CFTR gene Mutation			0.4
508del Homozygous (%)	26	24	
508del Heterozygous (%)	36	24	
Other/Unknown (%)	38	52	
Pancreatic insufficiency (%)	79	86	0.3
High dose of pancreatic enzymes supplements (%)	32	38	0.6
BMI (kg/m^2^) (mean ± SD)	22.1 ± 3.1	20.9 ± 2.9	0.1
BMI < 20 (kg/m^2^) (%)	24	48	0.03
FEV_1_% (mean ± SD)	62 ± 22	52 ± 22	0.05
FEV_1 _< 40% (%)	19	35	0.2
Airway colonization (%)	95	84	0.2
Pseudomonas colonization (%)	61	43	0.1
NTproBNP (pg/ml, mean ± SD)	57 ± 45	573 ± 1933	0.02
LVEF (%)	65 ± 6	59 ± 12	0.03
Type of LV dysfunction			0.01
Systolic (%)	2	14.5	
Isolated diastolic (%)	6	14.5	
No dysfunction (%)	92	71	
Average longitudinal 2C Strain	−21.4 ± 3	−19.4 ± 5	0.02
Average circumferential Strain	−20 ± 4	−19 ± 5	0.5

BMI, body mass index; FEV_1_, forced expiratory volume in the first second; NTproBNP, N-terminal pro B-type natriuretic peptide; LVEF, left ventricular ejection fraction; LV, left ventricle; 2C, two-chamber longitudinal strain.

## 4 Discussion

**Figure 3:**
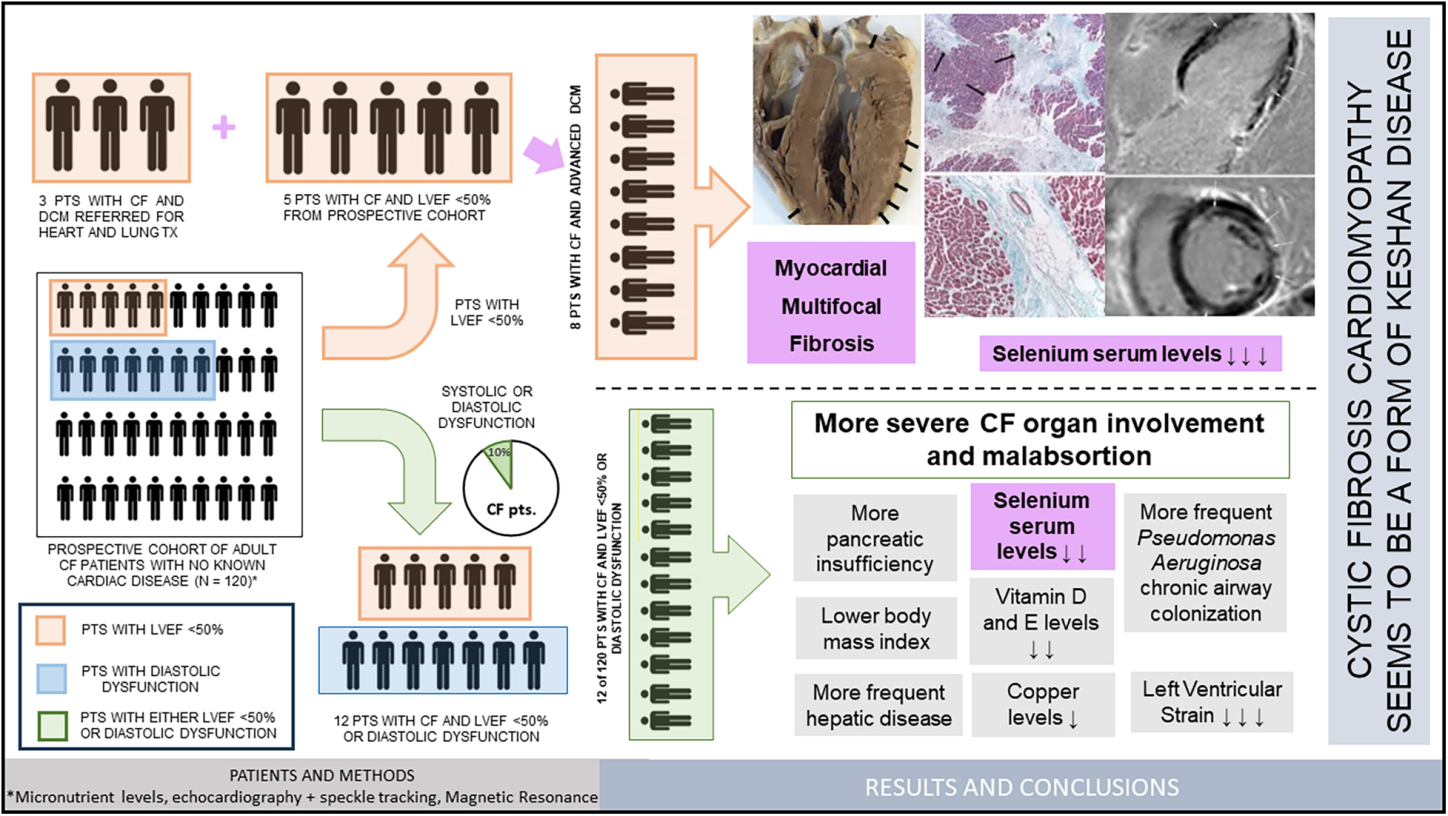
The coincidence of severe cystic fibrosis and cardiomyopathy in 3 patients referred to us for heart and lung transplantation suggested the existence of a specific type of cardiomyopathy related to cystic fibrosis. We recruited a prospective cohort of 120 patients with cystic fibrosis who were studied with echocardiogram and laboratory tests. Patients with LVEF < 50% (5 from the prospective cohort plus the 3 initial patients) underwent cardiac magnetic resonance, where a patchy pattern of late gadolinium enhancement was identified. Selenium deficiency was more frequent among these patients. Pathologic study of the explanted hearts from the 3 transplanted patients showed a distinct pattern of multifocal myocardial fibrosis similar to that of Keshan disease and other entities with nutrient malabsorption. Within the prospective cohort, we compared the clinical and laboratory findings of patients with significant diastolic or systolic dysfunction (*n *= 12) with those of cystic fibrosis patients with normal myocardial function (*n* = 108). Patients with cardiac dysfunction showed more severe organ involvement and lower levels of Selenium, other trace elements and several nutritional parameters. We conclude that a distinct type of cardiomyopathy similar to that of Keshan disease is present in a small percentage of patients with cystic fibrosis, and is associated with lower selenium levels, poorer nutritional status and more severe organ involvement.

Our investigation delineates a unique subset of cardiomyopathy in adult patients with cystic fibrosis, characterized by a distinct pattern of multifocal myocardial fibrosis marked by characteristic histological findings and corroborating CMR imaging, with an observed association with selenium deficiency. In our CF population, the incidence of left ventricular dysfunction of any form is approximately 10%, with overt CMP presenting in about 4% of cases, usually associated with more severe pulmonary and nutritional manifestations of CF. These outcomes are summarized in Figure 3.

Historically, the exploration into cardiac manifestations in CF traces back to the 1980s when cor pulmonale was frequently cited as a cause of mortality within this demographic, and non-invasive diagnostic options remained equivocal. There exists a paucity of contemporary data delineating the prevalence and characteristics of cardiac involvement in CF, partly because earlier studies emerged from an era when the clinical prognosis was graver and long-term survival past adolescence was rare (15–18).

The cohort of CF patients with cardiomyopathy in our study exhibited clinical, electrocardiographic, radiological, and echocardiographic features commonly associated with other forms of dilated cardiomyopathy. Nonetheless, histological analyses in all four patients where such data were attainable consistently revealed the distinctive presentation of patchy myocardial fibrosis (MMF). This discovery bears a connection to necropsy findings from the 1970s in children with CF. Notably, Oppenheimer and colleagues flagged a prevalence of MMF, then referred to as multifocal myocardial necrosis, at 4% among 143 autopsies of children with CF who had succumbed to various etiologies, including acute heart failure (6).

The observation of late gadolinium enhancement in four out of the five CF CMP cases subject to CMR imaging suggests substantial myocardial fibrosis. Particularly notable is the patchy intramyocardial distribution of this uptake in three instances, which diverges from patterns characteristic of ischemic and other cardiomyopathies. This distribution may indeed be reflective of the histological findings we observed. Such CMR findings have not been previously documented in CF patients and could potentially serve as a non-invasive marker for the diagnosis of this specific cardiomyopathy variant.

Nezelof et al. (8) previously identified MMF in 16 instances from an extensive collection of necropsy studies in children younger than two years. Over two decades later, the same researchers noted a resemblance between this fibrosis pattern and Keshan disease, suggesting a shared nutritional deficit as the etiological basis (9). More recently, selenium deficiency has been linked to Keshan disease, and dietary selenium supplements have proven instrumental in its mitigation and prevention (11, 12). In 2000, Zebrak et al. (7) conducted research on a cohort of 18 children with CF who experienced sudden fatality, finding the defining MMF pattern in the vast majority (89%). Here, the possibility of the F508del CFTR gene mutation as a predisposing factor was contemplated. Collectively, these studies have hypothesized a potential nutritional deficiency as a contributory factor in cardiac involvement, although subsequent research to substantiate this postulation has been lacking.

Prior surveys of cardiac function in CF patients have reported a wide range of incidences for right and left ventricular dysfunction, from nonexistent to more than 40% (5, 15–18) The investigation of diastolic LV dysfunction by Johnson et al. (17) and Koelling et al. (18) highlighted a common observation — the absence of a correlation between LV dysfunction and right ventricular complications, poised alongside a correlation of LV dysfunction with the severity of pulmonary disease and clinical CF evaluations.

The diagnostic methods for detecting LV dysfunction have seen significant advancements in recent times (5, 18–23). Concurrently, substantial enhancements in CF treatment have resulted in improved survival rates and elevated quality of life for patients (2). Accordingly, the prominence of cardiovascular symptoms associated with CF is likely to become increasingly salient in the foreseeable future (3, 22).

Myocardial deformation analysis, utilizing echocardiographic speckle-tracking techniques, has earned validation as an effective screening modality for subclinical cardiomyopathy across diverse etiologies. The detection of reduced strain and strain-rate values among CF patients with standard echocardiographic results dates back to 2011 and is corroborated by our research (19–21, 24). Thus, our data supports the growing consensus regarding the utility of myocardial deformation analysis as a sensitive method for uncovering incipient cardiac conditions within the CF population (20, 21, 24–26).

Out of the numerous vitamins and trace elements analyzed in our study, selenium (Se) deficiency has consistently emerged as the singular nutritional deficiency notably linked with various facets of myocardial dysfunction. This deficiency is not only associated to a 14.5% occurrence of CMP but is also identified as a factor contributing to diminished global left ventricular ejection fraction, altered myocardial deformation indices, and heightened levels of NT-proBNP. These relationships appear to follow a non-linear trend, analogous to what is observed in essential element-deficiency disorders, where damage typically manifests only below a cut-off (deficiency) value. The presence of normal Se levels in three out of eight CF patients diagnosed with CMP in this series could be attributed to delayed CF diagnosis in two of the individuals, positing that prolonged malnutrition—resulting from untreated CF-related digestive issues—may have led to exposure to Se deficiency and subsequent myocardial injury. Additionally, genetic variables may exert a significant role in determining the impact of selenium deficiency among patients. Future investigations are anticipated to elucidate the reparative potential of selenium supplementation in CF patients afflicted by low selenium-associated cardiomyopathy.

In 2012, Frustaci et al. documented a group of patients who developed dilated CMP subsequent to intestinal bypass surgery. Notably, after six months of intravenous selenium (Se) supplementation, the majority demonstrated a marked improvement in myocardial contractility (27).

Antioxidants play a crucial role in mitigating the harmful impacts of cellular oxidation resulting from free oxygen radicals (28–30). Selenium is an integral component of selenoproteins, such as glutathione peroxidases, and confers protection against oxidative stress-induced damage (22, 28, 30, 31–33). Moreover, particular signaling pathways and their interaction with microRNAs, as well as autophagy processes, contribute to the antioxidative effects of these proteins (34, 35). Deficiencies in micronutrients can profoundly affect mitochondrial energy production, endothelial function regulation, skeletal muscle protection, and thyroid metabolism (36–38). The link between selenium deficiency and cardiomyopathy has been established in humans, as seen in Keshan disease, and among animals, as illustrated by the condition known as “white muscle disease” (30, 31).

CF is characterized by a pronounced chronic inflammatory response and elevated levels of oxidative stress (22). Proper function of the CF transmembrane conductance regulator (CFTR) appears to shield myocytes from ischemia/reperfusion injury (39). Contemporary studies reveal adult CF patients still exhibit total selenium levels, both organic (Se-cysteine) and cationic forms of the element, that are significantly lower compared to healthy individuals (40). Case reports linking CF, CMP, and selenium deficiency corroborate this finding, lending support to the hypothesis of a relationship between these conditions (41).

A recent meta-analysis on the effect of supplementation with antioxidant principles in patients with CF suggested a relationship between serum selenium levels and respiratory function parameters such as FEV_1_. The different studies confirmed the positive correlation between serum selenium levels and antioxidant status described in the literature. The effect of supplementation on clinical variables is more controversial. The lack of efficacy of supplementation could be due to short treatment and follow-up periods in an entity with poorly understood pathophysiology (42, 43).

There is a growing consensus that even CF patients who adhere to current nutritional guidelines may experience micronutrient shortfalls that are associated with poorer clinical outcomes (44). Selenium supplementation must be judiciously administered, as elevated levels are linked to adverse consequences (45, 46). Future research is essential for elucidating the underlying mechanisms of cardiomyopathy tied to selenium deficits.

Limitations of the present study warrant careful interpretation when applying these findings across different populations and periods. As a cross-sectional analysis, it cannot establish causative links.

In conclusion, our investigation identified a particular form of cardiomyopathy in CF adults, which may be considered a variant analogous to Keshan disease. Diagnosis of this cardiomyopathy is attainable via non-invasive methods, and proactive screening using myocardial deformation echocardiographic techniques is viable. We observed that significant cardiac impairment occurs in 10% of a non-selected adult CF cohort, with half exhibiting systolic dysfunction. Notably, a correlation between CF CMP and reduced selenium levels was determined, highlighting a potential avenue for preventative measures and therapeutic intervention. Continued research is required to further elucidate the pathophysiological relationship between cardiomyopathy and selenium deficiency.

## Data Availability

The raw data supporting the conclusions of this article will be made available by the authors, without undue reservation.
