# Supplementary material for: The cardiomyopathy of cystic fibrosis: a modern form of Keshan disease
Source: Front Cardiovasc Med. 2024 Feb 1;11:1285223. doi: 10.3389/fcvm.2024.1285223 (PMC10867141; doi:10.3389/fcvm.2024.1285223)
Supplement: Supplementary file 1 [file Datasheet1.docx]

Supplementary Material

**Appendix I.** Method for processing of trace element samples:

Contamination in the collection and handling of samples was avoided in accordance with the Guideline of the Clinical and Laboratory Standards Institute (accessible at:

http://shop.clsi.org/c.1253739/site/Sample_pdf/C38A_sample.pdf).

The last sample was collected in trace element-free tubes (Vacutainer®, Becton Dickinson). Whole blood was collected in ethylene-diamine tetra acetic acid (EDTA) tubes (Vacutainer®, Becton Dickinson).

Serum selenium (μg/L), blood lead (μg/dL) and blood cadmium (μg/L) concentrations were determined by electrothermal atomic absorption spectrometry with Zeeman background correction on a Perkin Elmer AAnalyst 800. Serum copper (μg/dL) and zinc (μg/dL) were measured by flame absorption spectrometry on a Perkin Elmer AAnalyst 200. Blood mercury (μg/L) was measured in a direct manner without carrying out the digestions in a Perkin Elmer SMS-100 based on the EPA 7473 method: thermal decomposition, catalytic reduction, amalgamation, desorption and atomic absorption spectrometry. Internal quality controls (Seronorm® trace elements) were assessed in every series of samples to check the reproducibility and accuracy of the measurements. The lower limits of detection were 0.2 μg/dL for blood lead,0.1 μg/L for blood cadmium and 0.3 μg/L for blood mercury. For patients with either one of blood lead, cadmium or mercury below the limits of detection, a level equal to the limit of detection divided by the square root of 2 was imputed. Data from the Trace Elements Laboratory of Hospital Clínico San Carlos were used to establish the normal ranges for all the metals tested in our group.

**Appendix II.** Echocardiographic measurements:

Echocardiographic evaluation was performed with a IE33 digital ultrasound system®, (Philips Medical Systems, Amsterdam, Holland) using a S5-1 transducer, and followed the recommendations of the American Society of Echocardiography (ASE).**^w1,w2^**

Echocardiographic measurements were performed according to the American Society of Echocardiography (ASE) recommendations.w1 LV end-diastolic diameter, atrial dimensions, and interventricular septal width were measured with M mode from left parasternal view. Global left ventricular function was assessed from an apical four-chamber view by measuring LV ejection fraction (LVEF) using biplane Simpson’s rule. M-mode measurement of tricuspid annulus plane systolic excursion was calculated (TAPSE), with the M- mode cursor aligned through the tricuspid annulus in the apical 4-chamber view.

Pulsed-wave Doppler: The mitral Doppler signal was recorded in the apical four-chamber view with the sample volume placed at the tip of the mitral valve. Doppler measurements were done following the ASE recommendations.**^w2^** Peak early (E) and late (A) transmitral filling velocities and their ratio (E/A), deceleration time of E (DTE), and isovolumic relaxation time (IVRT) were measured from mitral inflow velocities. Pulsed tissue Doppler data from mitral and tricuspid annulus were recorded by placing a tissue Doppler sample volume at the septal and lateral annulus in the apical four-chamber view. Peak systolic (S´) and early diastolic (E´) velocities were recorded and the E/E´ mitral flow ratio was calculated. The Tei index of LV was calculated by DTI as the sum of isovolumic relaxation and isovolumic contraction time divided by ejection time.

LV longitudinal and circumferential strain measurements were performed by speckle tracking technique according to the EACVI/ASE recommendations.**^w3^**

For speckle-tracking analysis, cine-loops from two standard LV apical views (4-chamber and 2- chamber) and basal short axis view were recorded using gray scale harmonic imaging, with the highest possible frame rates (between 55-90 frames/sec). Care was taken to obtain the best LV visualization and the more reliable delineation of its endocardial border in each view. Three consecutive heart cycles were recorded and averaged. The analysis of recorded files was performed offline by two experienced blinded echocardiographers using commercially available semiautomated two-dimensional strain software (QLAB 7.0; Philips Medical Systems software). The endocardial borders were traced semiautomatically by manually marking two positions on both sides of the mitral annulus and one at the apex, allowing automatic tracing in the end-diastolic frame of the 2D images, and the operator manually adjusted the region of interest in segments that failed to track properly. Any segments that subsequently failed to track were excluded. Echocardiogram was excluded for the analysis when more than 2 segments were invalid.

A series of 21 healthy adults matched for age and gender served as a control group for strain and strain-rate data comparisons.

A reproducibility analysis of myocardial deformation measures was performed by two different observers who analyzed a group of 20 patients from the study. Measures were repeated after an interval of 6 weeks by both echocardiographers to assess the intra- and inter-observer agreement. For longitudinal strain measurements, intraclass correlation coefficient (ICC) was 0.84 (0.47-0.96; p<0.001) for the intra-observer analysis, while ICC was 0.71 (0.40-0.87; p<0.001) for the inter-observer comparison. For circumferential strain measurements, ICC was 0.89 (0.64-0.97; p<0.001) for the intra-observer analysis, and 0.70 (0.38-0.87; p<0.001) for the inter-observer comparison.

References for appendix II:

w1. Lang RM, Badano LP, Mor-Avi V, Afilalo J, Armstrong A, Ernande L, et al. Recommendations for cardiac chamber quantification by echocardiography in adults: An update from the American Society of Echocardiography and the European Association of Cardiovascular Imaging. J Am Soc Echocardiogr 2015; 28:1-39

w2. Quiñones MA, Otto CM, Stoddard M, Waggoner A, Zoghbi WA. Recommendations for quantification of Doppler echocardiography: a report from Doppler Quantification Task Force of the Nomenclature and Standards Committee of the American Society of Echocardiography. J Am Soc Echocardiogr 2002; 15:167-184.)

w3. Voigt JU, Pedrizzetti G, Lysyansky P, Marwick TH, Houle H, Baumann R, et al. Definitions for a Common Standard for 2D Speckle Tracking Echocardiography: Consensus Document of the EACVI/ASE/Industry Task Force to Standardize Deformation Imaging. J Am Soc Echocardiogr 2015; 28:183-93)

**Appendix III.** Cardiac magnetic resonance procedure:

CMR was performed with a 1.5T equipment, (Philips Intera CV, Best, The Netherlands®) in CF patients with established CMP and in a subgroup of 20 CF individuals without cardiac dysfunction. CMR protocol is described in appendix III of supplemental material.

Cardiac synchronization was obtained with ECG signal obtained through 4 electrodes. A stack of short axis cine views covering the left ventricle from base to apex was acquired for the determination of LV mass. For cine imaging, a steady-state free-precession sequence with retrospective gating combined with sensitivity encoding was used (>25 phases/cardiac cycle; repetition time, 2.7 ms; echo time, 1.4 ms; flip angle, 60°) during an end-expiratory breath hold of ≈5 seconds. Typical in-plane spatial resolution was 1.8×1.8 mm with a slice thickness of 8 mm. Perfusion imaging was performed using an intravenous bolus of 0.2 mmol/kg Gd-DTPA (Magnevist, Bayer, Berlin, Germany®) at an injection rate of 3 ml/s, followed by a flush of 20 ml of saline solution at the same rate. Delayed enhancement imaging was carried out 10 to 15 minutes after Gd-DTPA bolus injection. Look-Locker sequence guided us to find the optimal inversion time in delayed enhancement imaging.
